# Characterization of Calflagin, a Flagellar Calcium-Binding Protein from *Trypanosoma congolense*

**DOI:** 10.1371/journal.pntd.0004510

**Published:** 2016-04-07

**Authors:** Brett A. Eyford, Laura Kaufman, Orly Salama-Alber, Bianca Loveless, Matthew E. Pope, Robert D. Burke, Enock Matovu, Martin J. Boulanger, Terry W. Pearson

**Affiliations:** 1 Department of Biochemistry and Microbiology, University of Victoria, Victoria, British Columbia, Canada; 2 College of Veterinary Medicine, Animal Resources and Biosecurity, Makerere University, Kampala, Uganda; US Food and Drug Administration, UNITED STATES

## Abstract

**Background:**

Identification of species-specific trypanosome molecules is important for laboratory- and field-based research into epidemiology and disease diagnosis. Although *Trypanosoma congolense* is the most important trypanosome pathogen of cattle in Africa, no species-specific molecules found in infective bloodstream forms (BSF) of the parasites have been identified, thus limiting development of diagnostic tests.

**Methods:**

Immuno-mass spectrometric methods were used to identify a protein that is recognized by a *T*. *congolense*-specific monoclonal antibody (mAb) Tc6/42.6.4. The identified molecule was expressed as a recombinant protein in *E*. *coli* and was tested in several immunoassays for its ability to interact with the mAb. The three dimensional structure of the protein was modeled and compared to crystal- and NMR-structures of the homologous proteins from *T*. *cruzi* and *T*. *brucei* respectively, in order to examine structural differences leading to the different immunoreactivity of the *T*. *congolense* molecule. Enzyme-linked immunosorbent assays (ELISA) were used to measure antibodies produced by trypanosome-infected African cattle in order to assess the potential for use of *T*. *congolense* calflagin in a serodiagnostic assay.

**Results:**

The antigen recognized by the *T*. *congolense*-specific mAb Tc6/42.6.4 was identified as a flagellar calcium-binding protein, calflagin. The recombinant molecule showed immunoreactivity with the *T*. *congolense*-specific mAb confirming that it is the cognate antigen. Immunofluorescence experiments revealed that Ca^2+^ modulated the localization of the calflagin molecule in trypanosomes. Structural modelling and comparison with calflagin homologues from other trypanosomatids revealed four non-conserved regions on the surface of the *T*. *congolense* molecule that due to differences in surface chemistry and structural topography may form species-specific epitopes. ELISAs using the recombinant calflagin as antigen to detect antibodies in trypanosome-infected cattle showed that the majority of cattle had antibody responses. Area under the Receiver-Operating Characteristic (ROC) curves, associated with host IgG and IgM, were calculated to be 0.623 and 0.709 respectively, indicating a positive correlation between trypanosome infection and the presence of anti-calflagin antibodies.

**Conclusions:**

While calflagin is conserved among different species of African trypanosomes, our results show that *T*. *congolense* calflagin possesses unique epitopes that differentiate this protein from homologues in other trypanosome species. MAb Tc6/42.6.4 has clear utility as a laboratory tool for identifying *T*. *congolense*. *T*. *congolense* calflagin has potential as a serodiagnostic antigen and should be explored further for its utility in antigen-detection assays for diagnosis of cattle infections.

## Author Summary

African trypanosomes are parasites that infect humans and domestic animals, causing severe socioeconomic distress in sub-Saharan Africa. Thus developing tools for laboratory- and field-based research for application to epidemiology and disease diagnosis is important if the diseases caused by these parasites are to be controlled. Although *Trypanosoma congolense* is the most important trypanosome pathogen of cattle in Africa, no species-specific molecules found in infective bloodstream forms (BSF) of the parasites have been identified, thus limiting development of diagnostic tests and epidemiological tools. We have biochemically characterized and modeled the structure of one such molecule, called calflagin, from this parasite and genetically engineered and purified a form of the protein for use in testing cattle for trypanosome infections. In addition, we made new monoclonal antibodies to the calflagin molecule. Our results show that the calflagin and its specific antibodies are useful tools for research in epidemiological and diagnostic applications.

## Introduction

Of the major trypanosome species that infect cattle, *Trypanosoma brucei*, *T*. *vivax* and *T*. *congolense*, the latter has received much attention recently because all four of its major life-cycle stages can be cultured *in vitro*, making it an ideal parasite for study [1,2]. *T*. *congolense* is widespread and considered the most important cattle pathogen, but also infects sheep, pigs, goats, horses and camels. The parasites cause a chronic wasting (cachexia) in cattle, characterized by anemia, weight loss and immunosuppression. The disease is fatal if untreated, and causes severe socioeconomic problems in sub-Saharan Africa.

To improve disease control it is important to develop tests that can specifically detect *T*. *congolense*. Although several *T*. *congolense*-specific molecules have been described in forms of the parasites that reside in the tsetse insect vector, including glutamic acid-alanine rich protein (GARP; [3]), protease resistant surface glycoconjugate (PRS; [4]), *congolense* epimastigote specific protein (CESP; [5]) and *c**ongolense* insect stage surface antigen (CISSA; [6]), these are not expressed in *T*. *congolense* bloodstream forms (BSF) and thus are not useful for detection of infections in animals. Monoclonal antibodies (mAbs) specific for *T*. *congolense* have been previously described [7] and used to develop antigen-detection assays for detection of *T*. *congolense* infected cattle [8], but the relevant antigens were not identified. Another *T*. *congolense*-specific mAb, Tc6/42.6.4, was derived many years ago and was shown to bind to non-surface molecules [9]. This mAb showed very strong specific binding to *T*. *congolense* procyclic culture forms (PCF) and to BSF as determined in various immunoassays, and has thus been a useful laboratory tool for identification of *T*. *congolense*. However, several attempts over the past 25 years to identify its cognate antigen were unsuccessful, thus limiting the utility of the molecule in development of diagnostic tests.

Here we describe the identification and immunological and biochemical characterization of the molecule recognized by the *T*. *congolense*-specific mAb Tc6/42.6.4. An immuno-mass spectrometric approach was used to identify the antigen as *T*. *congolense* flagellar calcium-binding protein (FCaBP), also called calflagin. Recombinant *T*. *congolense* calflagin was expressed and shown to react strongly with mAb Tc6/42.6.4. The recombinant molecule was used as immunogen to derive several new mAbs, some of which were *T*. *congolense*-specific, whereas others bound to epitopes also found in *T*. *brucei*, showing that there are species-specific and common epitopes of calflagin. Molecular modeling and structural comparison with calflagins from *T*. *b*. *brucei* and *T*. *cruzi* revealed four non-conserved regions on the surface of the *T*. *congolense* calflagin molecule that could serve as species-specific epitopes, due to their alteration in surface chemistry and structural topography. The recombinant protein was tested for its potential as a serodiagnostic antigen for detection of antibodies produced by cattle infected with *T*. *congolense*.

## Materials and Methods

### Trypanosomes and cell culture

*T*. *congolense* IL3000 (savannah strain [10]), *T*. *congolense* K45/1 (Kilifi strain [11]) and *T*. *simiae* CP-11 [12] were originally obtained as cryopreserved BSF stabilates from the International Livestock Research Institute, formerly the International Laboratory for Research on Animal Diseases, Nairobi, Kenya. PCF trypanosomes were produced by transformation of BSF [13] and were maintained in culture at 27°C in minimal essential medium containing 10% heat-inactivated fetal bovine serum (PCF medium) as previously described [3]. Lysates of the four major life cycle stages of *T*. *congolense* (bloodstream, procyclic, epimastigote and metacyclic forms) were obtained from Dr. Noboru Inoue (Obihiro University, Hokkaido Japan) as part of our collaborative work on *T*. *congolense* protein expression [1].

### Monoclonal antibody Tc6/42.6.4

The hybridoma secreting monoclonal antibody Tc6/42.6.4 was derived from a BALB/c mouse that were immunized with intact, irradiated *T*. *congolense* BSF [9]. The mAb is an IgG_2b_, kappa and was used as diluted murine ascites fluid. The mAb was originally reported to recognize a proteinase K sensitive antigen in *T*. *congolense* and showed no reactivity with *T*. *brucei* sspp., *T*. *vivax* or *Leishmania braziliensis* [9]. Thus the mAb appears to be specific for a *T*. *congolense*-specific epitope on a protein of approximately 26–31 kDa in both *T*. *congolense* BSF and PCF, as determined by immunoblotting and immunofluorescence microscopy [9].

### Immunoenrichment of the antigen recognized by mAb Tc6/42.6.4

The molecule recognized by mAb Tc6/42.6.4 was immunoenriched from lysates of *T*. *congolense* PCF and identified by peptide mass fingerprinting using matrix-assisted, laser desorption ionization time of flight (MALDI-TOF) and electrospray ionization (ESI) tandem mass spectrometry (MS/MS) of trypsin-digested, polyacrylamide gel-separated protein bands. In brief, goat anti-mouse IgG Dynabeads (50 μL slurry; Cat. No. 110.33; Invitrogen, Oslo, Norway) were rinsed once with 50 μL sterile, ice cold phosphate buffered saline (PBS) and then resuspended in 500 μL PBS. Ten μL of Tc6/42.6.4 ascites fluid were added and mixed end-over-end overnight at 4°C. The beads were magnetically pelleted and washed 3 times with sterile PBS before incubation with trypanosome lysates prepared as follows: *T*. *congolense* IL3000 PCF (10^7^ cells) were pelleted (5 min at 10,000 g), washed once with sterile PBS, resuspended in 1 mL sterile PBS + 1x protease inhibitor cocktail V (Cat. No. 539137, Calbiochem, Darmstadt Germany) and lysed by sonication on ice. The immunoadsorbent bead-lysate mixture was mixed end-over-end overnight at 4°C. The magnetic beads were washed 3 times with 1 mL PBS/0.03% 3-[(3-cholamidopropyl) dimethylammonio]-1-propanesulfonate detergent. Bead-bound proteins were eluted in 50 μL 2 x Laemmli SDS sample buffer at 60°C for 15 minutes. Proteins in the eluate were separated by SDS-PAGE in parallel gel lanes and were analyzed by immunoblotting (see below) and by staining with colloidal Coomassie Brilliant Blue G250 [14]. The immunoblots were used to indicate which stained bands corresponded to the antigen recognized by mAb Tc6/42.6.4. The relevant gel bands were excised, the proteins were de-stained, reduced, alkylated and digested with trypsin as previously described [15]. Peptides were extracted and analyzed by mass spectrometry as described below.

### Gel electrophoresis and immunoblotting

Proteins were separated by SDS-PAGE followed by transfer to Hybond^TM^-P polyvinylidene difluoride (PVDF) transfer membrane (Cat. No. RPN303F; GE Healthcare, Little Chalfont, UK) as previously described [3]. The primary antibody (Tc6/42.6.4 ascites fluid) was diluted 1:2,000 and the secondary detection reagent used after the initial antibody pulldowns was a 1:2,000 dilution of horseradish peroxidase (HRP)-conjugated CleanBlot IP Detection Reagent (Cat. No. 21230, Thermo Scientific, Waltham, USA). Clean-Blot IP Detection Reagent is optimized for post-immunoprecipitation immunoblotting. The reagent specifically binds to properly folded primary antibodies (whole IgG) without also binding to fragments of the IP antibodies, which usually accompany the immunoprecipitated protein through electrophoresis and membrane transfer. The use of this reagent was necessary because the antigen of Tc6/42.6.4 (~26–21 kDa) migrates closely with the antibody light chain (~25 kDa). Once the antigen (calflagin) was identified, the secondary antibody used thereafter was a 1:20,000 dilution of HRP conjugated goat anti-mouse (Cat. No. 1858413; Pierce Chemical Co., Rockford, USA). The substrate used for all immunoblots was SuperSignal West Dura (Cat. No. 34075, Thermo Scientific, Waltham, USA) along with Kodak Biomax MR film (Cat. No. 353949, Eastman Kodak Company, Rochester, USA) to detect chemiluminescence. After development of the autoluminograms, proteins on the PVDF membrane were stained with 0.2% (w/v) nigrosin in PBS.

### Mass spectrometry

MALDI-TOF-MS/MS and ESI-MS/MS were performed at the UVic-Genome BC Proteomics Centre (Victoria, BC) to analyze peptides in the tryptic digests of gel bands. An Applied Biosystems MDS Sciex TOF/TOF 4800 Mass Analyzer was used (Concord, Canada). The 25 most intense peaks in the mass range of 800–4000 m/z were selected for MS/MS fragmentation. ESI-MS/MS analyses were performed using an Applied Biosystems/MDS Sciex QSTAR Pulsar I Hybrid Quadrupole-TOF LC-MS/MS Mass Spectrometer. MS/MS spectra were acquired by selecting the top 2 most intense eluting ions in the 400–1600 *m/z* range with a 2+ to 4+ charge state. MALDI-TOF/TOF and MS/MS data were searched against a *T*. *congolense* proteome database (January 2009 version; ftp.sanger.ac.uk/pub/project/pathogens/Trypanosoma/congolense/).

### Gene cloning and recombinant protein expression

Based on results from the MS experiments, the gene encoding calflagin was cloned and expressed in *E*. *coli*. To obtain the full-length gene, the primers 5’-GGCTCATATG GGT TGC TCT GGA TCA A-3’ and 5’-CGCGGATCC CTA TCA GTG GTA GGG GTC T-3’ were used. Live *T*. *congolense* IL3000 PCF were used as the source of the template DNA. The PCR product (634 bp encoding 204 amino acids) was cloned into the vector pET-24a via NdeI/BamHI restriction sites and chemically transformed into *E*. *coli* TOP10. Plasmid insert sequence was verified by standard dideoxy sequencing (Eurofins MWG/Operon, Huntsville USA). The selected insert was identical to two of the four *T*. *congolense* calflagin open reading frames (ORFs; TcIL3000.0.43820 and TcIL3000.8.5280). For protein expression, the plasmid was inserted into *E*. *coli* BL21*DE3. The bacteria were grown to log phase in LB broth and calflagin expression was induced with 1 mM isopropyl β-D-thiogalactopyranoside (IPTG) or with auto-induction medium as previously described [16]. The expressed calflagin was detected by immunoblotting using mAb Tc6/42.6.4 as described above.

An N-terminal hexa-histidine tagged version of calflagin (Ser12–Pro202) was also expressed. A PCR product using the primers 5’-CGTCATATG TCC AAG GGC TCT GCG TG-3’ and primer 5’-GCTGGATCC CTA GGG GTC TCC GAA CGC-3’ was cloned into pET-28a by NdeI/BamHI sites. This construct was made so that the protein could be purified from *E*. *coli* lysate for use in surface plasmon resonance (SPR) assays, for immunization of mice and derivation of new mAbs and for serodiagnosis experiments. The recombinant fragment was purified as previously described [16]. The His tag was not removed by thrombin cleavage because the protease buffer requires Ca^2+^ which is known to alter the folding state of calflagin [17–19] and which, in our experience, can cause protein precipitation. Five mM EDTA was added to all buffers to chelate trace calcium.

### Enzyme-linked immunosorbent assay

Indirect ELISAs were performed on parasite lysates or on recombinant molecules essentially as previously described [20]. Briefly, parasite lysates from the equivalent of 5 x 10^5^ cells per well or purified recombinant protein at 1 μg/ well were dried onto polystyrene ELISA plates. Tc6/42.6.4 ascites fluid (1:1,000 dilution) was used as a source of primary antibody. The secondary antibody was goat anti-mouse IgG/M (H+L)—alkaline phosphatase at 1:2,000 dilution (Cat. No. 31328, Thermo Scientific, Rockford USA). Substrate (para-nitrophenylphosphate) cleavage was measured by absorbance at 405 nm.

### Immunofluorescence and confocal microscopy

One mL (~10^6^ cells) of log-phase *T*. *congolense* IL3000 PCFs were pelleted (1 min at 10,000 g) and washed once with ice cold, sterile PBS. Pelleted cells were fixed for 10 min at room temperature (RT) by resuspending in 1 mL of either 4% paraformaldehyde in PBS or 1 part formalin to 9 parts PEM buffer (100 mM PIPES, 5 mM EGTA, 2 mM MgSO_4_, 0.2% Triton X-100, pH 6.8). The fixed parasites were pelleted and blocked with 1 mL of Super Block Blocking Buffer (Cat. No. 37515, Thermo Scientific, Rockford USA) + 0.3% Triton X-100. Fifty μL of the fixed cells were added to poly-L-lysine coated microscope slides and allowed to adhere for 15 min at RT. Primary antibodies were a combination of 1:1,000 dilution of Tc6/42.6.4 ascites fluid and a 1:500 dilution of rat antiserum specific for trypanosome paraflagellar rod protein [21]. Primary antibodies were incubated with parasites overnight at 4°C. Slides were washed three times by 5 minute immersions in PBS. The secondary antibodies, goat anti-mouse IgG (H+L)–Alexa Fluor 488 (Cat. No. A11029, Invitrogen, Eugene USA) and goat anti-rat-AlexaFluor 568 (Cat. No. A11077, Invitrogen, Eugene OR) were diluted 1:1,000 in blocking buffer and 50 μL were added to the slides for 1 hr at RT followed by washes as described above. Specimens were mounted on glass slides in Slow-Fade Gold with DAPI for visualization of DNA (Cat. No. S36938, Invitrogen, Eugene, OR, USA) and examined with a Zeiss LSM 700 confocal laser scanning microscope using a Zeiss 63 x oil-immersion objective lens (NA = 1.3). A series of optical sections of specimens were collected. Individual optical sections or maximum intensity projections were prepared using Zen software (2009, version 5.05.00; Carl Zeiss, Canada).

### Surface plasmon resonance analysis of mAb Tc6/42.6.4 binding

A Biacore 3000 surface plasmon resonance (SPR) instrument (GE Healthcare) was used to determine the kinetics of binding of mAb Tc6/42.6.4 to recombinant *T*. *congolense* calflagin. A high-resolution multi-concentration analysis was performed by first capturing mAb Tc6/42.6.4 using an affinity-purified sheep anti-mouse IgG antibody that was covalently coupled to the Biacore chip and then injecting the purified, recombinant calflagin at concentrations ranging from 62.5 to 500 nM. Data were double referenced and fit globally using a 1:1 Langmuir binding model. The general method has been described previously [22]. To further characterize the mAb, the variable regions of the genes encoding the antibody heavy and light chains from the hybridoma Tc6/42.6.4 were sequenced by Immunoprecise Antibodies Ltd. (Victoria, Canada).

### Homology model of *T*. *congolense* calflagin

A high confidence homology model of *T*. *congolense* calflagin (GeneDB accession TcIL3000.0.43820) was generated using the I-TASSER online server [23]. Coordinates of the Ca^+2^-free FCaBP structure from *Trypanosoma cruzi* (PDB code 3CS1) were used as the model template. The best predicted model was evaluated by a C-score of 0.99 (confidence score value which signifies a high quality of the predicted model), a TM-score of 0.85 ± 0.08 (which indicates a model of correct topology) generated by the I-TASSER server. Structural Figures were generated using PyMol [24]. Pairwise comparison of protein structures was performed using the DaliLite server [25].

### Collection of cattle blood and determination of trypanosome infection status

Field surveys were carried out in the trypanosomiasis endemic districts of Northern Uganda to identify areas suitable for obtaining blood from cattle. Blood was collected under supervision of a veterinarian from cattle jugular veins into EDTA Vacutainer tubes (Cat. No. 367841; Becton, Dickinson & Co., Franklin Lakes USA). From these, capillaries were prepared for the haematocrit centrifugation trypanosome enrichment technique [26] followed by light microscopy for detection of blood-borne parasites. From the EDTA treated blood, 500 μL portions were dispensed into 1.0 mL Eppendorf microcentrifuge tubes for future isolation of DNA. The remaining blood was centrifuged and plasma collected into cryovials that were immediately frozen in liquid nitrogen and stored at -80°C. As negative controls, blood and plasma were also collected from three calves born and kept at the Makerere University animal houses, with no exposure to trypanosomes or to tsetse insect vectors. All samples were stored in liquid nitrogen and were held at Makerere University.

Blood and plasma were taken from a total of 84 test animals. DNA was prepared from 100 μL of blood from each animal using a quick-gDNA miniprep kit (Cat. No. D3024, Zymoresearch, Irvine USA). To identify the infecting species, PCR for the Internal Transcribed Spacer (ITS) was performed as described by Njiru et al. [27]. For samples in which no signals were obtained, another run with a more sensitive nested PCR technique was performed [28].

### Measurement of anti-calflagin antibodies in sera from trypanosome-infected cattle

For the serodiagnostic ELISAs, each well of the microtitre plates was coated with 100 ng of recombinant *T*. *congolense* calflagin. ELISAs were performed as described above with the exception that the primary antibodies (cattle plasma) were diluted 1:100 and the secondary antibodies were sheep anti-bovine IgG or rabbit anti-bovine IgM, both conjugated with alkaline phosphatase and diluted 1:1,000. Each sample was tested in duplicate. Plasmas from the laboratory-raised calves were tested on each ELISA plate and used to define background signal. The average OD obtained using plasma from the 3 uninfected calves was subtracted from the average signal of each test sample. Diagnostic accuracy, which refers to the degree of agreement between a test and a reference standard, in this case between the presence of anti-calflagin IgM and IgG antibodies and parasitemia/PCR data for determination of trypanosome infection was calculated using a receiver operating characteristic (ROC) curve [29].

### Derivation of mAbs to recombinant calflagin

New calflagin-specific mAbs were derived by immunizing mice with purified recombinant *T*. *congolense* calflagin. Single step selection and cloning of hybridomas using ClonaCell-HY^TM^ medium (Cat. No. 3800, Stemcell Technologies, Vancouver Canada) was used as described previously [30]. A total of 1012 clone supernatants were tested in indirect ELISA using recombinant *T*. *congolense* calflagin as solid-phase adsorbed antigen.

## Results

### Species- and life cycle stage-specificity of mAb Tc6/42.6.4

MAb Tc6/42.6.4 was previously determined to be specific for *T*. *congolense* (subgenus *Nannomonas*) as it showed no reactivity with the two other major trypanosome species that infect African livestock, *T*. *brucei* sspp. (subgenus *Trypanozoon*) or *T*. *vivax* (subgenus *Duttonella*) [9]. To extend the characterization of mAb Tc6/42.6.4, lysates of *T*. *simiae* CP11 (subgenus *Nannomonas*) PCF were tested alongside lysates of *T*. *congolense* IL3000 PCF (subgenus *Nannomonas*; savannah strain) and *T*. *congolense* K45/1 (subgenus *Nannomonas*: Kilifi strain) for reactivity by indirect ELISA. Both savannah and Kilifi strains of *T*. *congolense* showed strong OD_405 nm_ s (2.3 and 2.8 respectively) whereas the *T*. *simiae* was negative (OD_405nm_ of 0.08).

**Fig 1:**
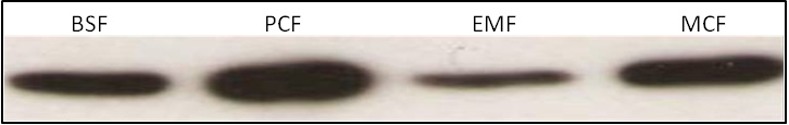
Immunoblot detection of antigen in the four major life cycle stages of *T*. *congolense* IL3000 using mAb Tc6/42.6.4. BSF: bloodstream forms; PCF: procyclic culture forms; EMF: epimastigote forms; MCF: metacyclic forms. Modified with permission from [1].

It was also previously reported that the antigen recognized by mAb Tc6/42.6.4 was present in both BSF and PCF of *T*. *congolense* [9]. To extend the life cycle stage specificity analysis, immunoblots were performed on all four life cycle stages of *T*. *congolense*. An immunoreactive band at ~26 kDa was observed in all four life cycle stages (bloodstream forms, procyclic forms, epimastigote forms and metacyclic forms) with procyclic and metacyclic forms showing the greatest intensity and epimastigote forms the lowest (Fig 1).

### Identification of the antigen recognized by mAb Tc6/42.6.4

**Fig 2:**
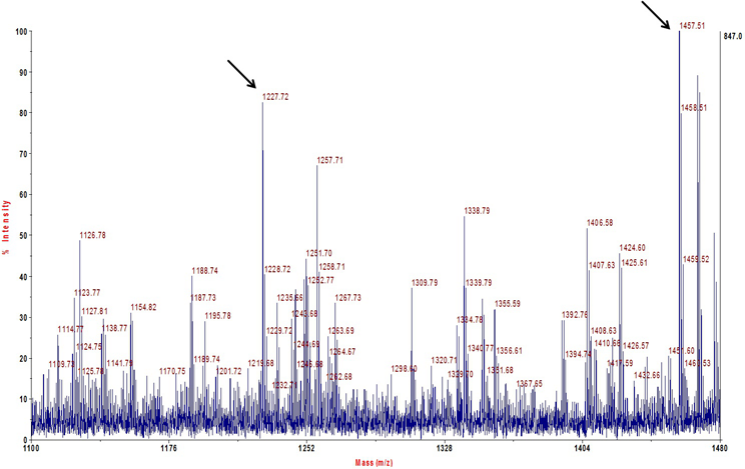
MALDI-TOF mass spectrum of the trypsin-digested ~26 kDa gel band recognized by mAb Tc6/42.6.4. The peak at 1227.72 m/z corresponds to the peptide VLQMHELTTR and the peak at 1457.51 m/z corresponds to the peptide LSFNEVCSGCER (with two carbamidomethylations).

**Fig 3:**
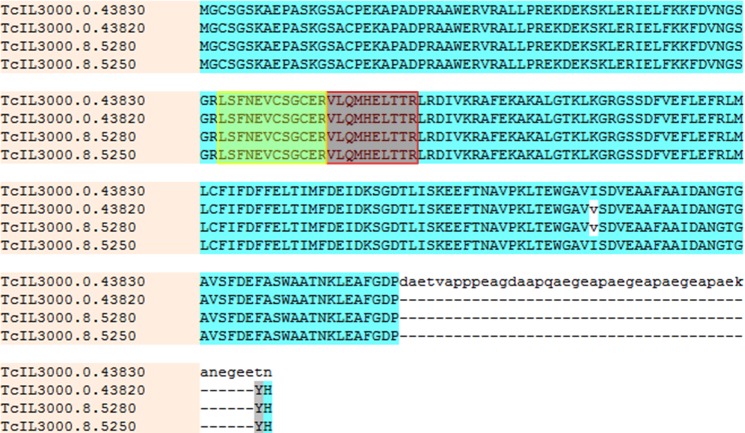
Multiple sequence alignment of the *T*. *congolense* calflagins. The positions of the two MS-identified tryptic peptides identified by mass spectrometry are highlighted in yellow and red boxes. The lower case, white highlighted v represent amino acid (valine-isoleucine) differences in two of the ORFs.

**Fig 4:**
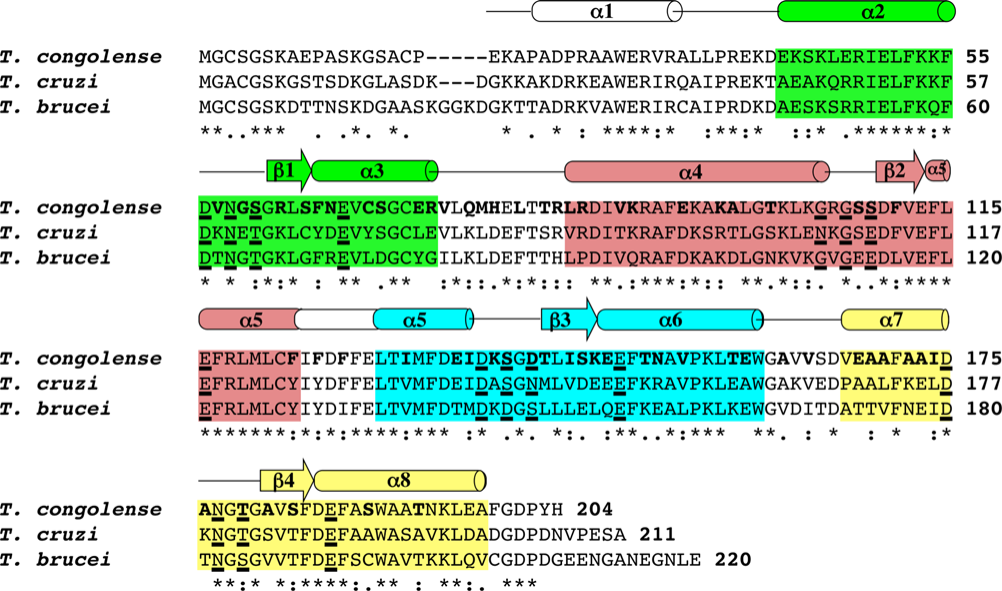
Primary sequence alignment of *T*. *congolense* calflagin with those of *T*. *cruzi* FCaBP and *T*. *brucei* Tb24. The secondary structural elements (α-helices and β-strands) are depicted as cones and arrows, respectively, and were derived from the I-TASSER based structure prediction for *T*. *congolense* calflagin, the x-ray crystal structure of *T*. *cruzi* FCaBP (3CS1) and the NMR data for *T*. *brucei* Tb24 (2LVV). The four EF-hands (EF1, EF2, EF3, and EF4) are highlighted in green, salmon, cyan, and yellow, respectively. Residues in the 12-residue Ca^2+^ binding loops at position 1, 3, 5 and 12 are underlined. Invariant basic residues on the protein surface that are associated with membrane binding are colored blue. Non-conserved surface-exposed residues are highlighted using bold print.

The antigen recognized by mAb Tc6/42.6.4 appears as a strong immunoreactive band of ~26 kDa when detected by immunoblotting on *T*. *congolense* lysates. After enrichment by pull-down with mAb Tc6/42.6.4, the antigen band at ~26 kDa was easily detected (S1 Appendix). However, Coomassie Brilliant Blue stained gels only showed a faint band at the antigen’s expected location (S1 Appendix). To identify the protein antigen, the ~26 kDa band was excised and subjected to in-gel trypsin digestion followed by peptide extraction and analysis by MALDI-TOF-MS/MS and ESI-MS/MS. Two peptides of trypanosome origin were identified by searching against the *T*. *congolense* proteome database (Fig 2). These two peptides, m/z 1227.72 and 1457.51 were determined by MALDI-TOF-MS/MS to have the sequences VLQMHELTTR and LSFNEVCSGCER respectively. The same peptides were also identified by LC-MS/MS. These two peptides were traced to proteins encoded by four, nearly identical, *T*. *congolense* ORFs, which at the time, were annotated as “undefined product” in the *T*. *congolense* database. Sequence alignment (Fig 3) of these was performed using the Muscle software program [31]. These proteins show strong homology with the Tb-24 family of flagellar calcium binding proteins (FCaBP or calflagin) from *T*. *brucei*, *T*. *cruzi* and other trypanosomatids. The *T*. *congolense* proteins showed 55% identity and 69% similarity to the *T*. *brucei* calflagin and showed 59% identity and 75% similarity to *T*. *cruzi* calflagin (Fig 4).

There are four ORFs in *T*. *brucei* designated Tb-17, Tb-24 (2 copies) and Tb-44. These are clustered on chromosome 8. The proteins encoded by Tb-17 and Tb-24 have predicted masses of ~24 kDa and Tb-44 of ~44 kDa. *T*. *congolense* shows a similar trend with one calflagin product longer than the other three. The larger Tb-44 calflagin shows a 20 kDa mass difference compared to the other *T*. *brucei* calflagins, whereas the difference in the *T*. *congolense* forms is only 4.2 kDa. The larger *T*. *congolense* calflagin is due to an extended C terminus, whereas most of the extra length in the *T*. *brucei* calflagin is due to an insertion in the middle of the protein. The *T*. *congolense* calflagins are also more similar to each other than the *T*. *brucei* versions. All four of the *T*. *congolense* ORFs are annotated as fully functional genes, however only two of the shorter versions (TcIL3000.8.5250 and TcIL3000.8.5280) have been traced to a chromosomal location (chromosome 8). The location of the remaining two hasn't been assigned yet.

*T*. *congolense* calflagin has been observed in all four major life cycle stages, as determined by iTRAQ-MS experiments [1]. In the current work, peptides were identified from the common region of calflagin (encoded by all four genes) and a peptide from the extended tail of the long version (TcIL3000.0.43830). Thus it is certain that at least the longer version is expressed.

### Cloning and expression of recombinant *T*. *congolense* calflagin

To confirm that the MS-identified protein was truly the antigen recognized by mAb Tc6/42.6.4, full length *T*. *congolense* calflagin (corresponding to the identical ORFs TcIL3000.0.43820 and TcIL3000.05280) was expressed as a recombinant protein in *E*. *coli*. A protein band corresponding to the expected size for recombinant calflagin was detected in Coomassie Brilliant Blue-stained gels after IPTG induction (S1 Appendix). The induced protein band also reacted with mAb Tc6/42.6.4 in immunoblots indicating that calflagin was in fact the cognate antigen recognized by this mAb (S1 Appendix).

### Characterization of *T*. *congolense* calflagin

**Fig 5:**
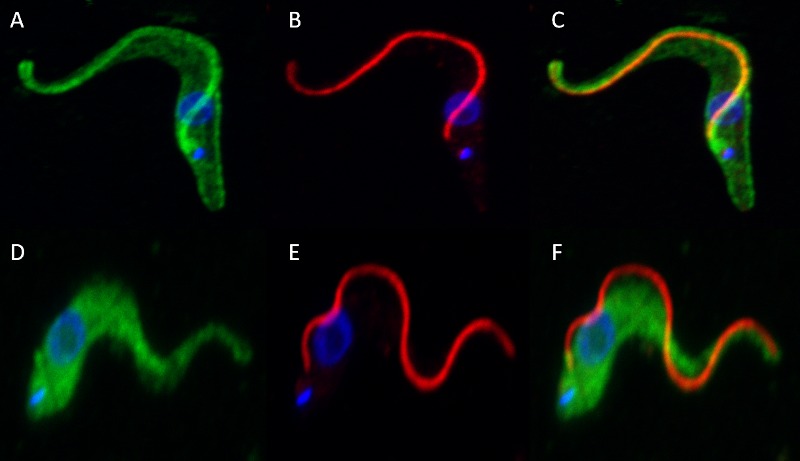
Confocal immunofluorescence microscopy showing localization of *T*. *congolense* calflagin in the presence and absence of calcium. Maximum intensity projections of fixed *T*. *congolense* PCF were probed with mAb Tc6/42.6.4 and anti-para-flagellar rod protein in the presence (A, B, and C) and absence (D, E, and F) of calcium. Green: calflagin; Red: para-flagellar rod protein; Blue: DAPI/DNA.

In *T*. *cruzi* and *T*. *brucei*, calflagin is thought to act as a calcium sensor that oscillates between the plasma membrane and the cytoplasm in response to intracellular Ca^2+^ concentration [32]. To test whether or not *T*. *congolense* calflagin displays similar calcium-induced localization, confocal immunofluorescence microscopy was performed using two different fixation protocols, one under normal cellular conditions and the other in the presence of a chelator (EGTA; Fig 5). In the presence of calcium, calflagin (green) is distributed throughout the cells but showed a marked increased intensity along the flagellum and the cell membrane (Fig 5A–5C). When incubated with a fixative containing a chelator, the cells showed only diffuse fluorescence in the cytoplasm and a complete lack of fluorescence along the flagellum and cell periphery (Fig 5D–5F).

### Structural properties of *T*. *congolense* calflagin

**Fig 6:**
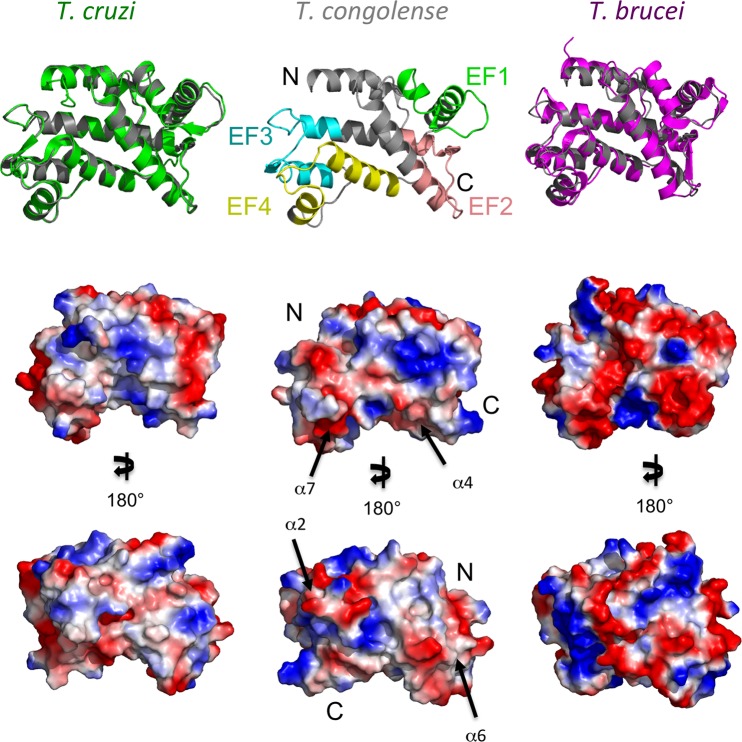
Structural characterization and surface analysis of *T*. *congolense* calflagin Modeled *T*. *congolense* calflagin (middle panel) was compared to the crystal structure of *T*. *cruzi* FCaBP (left vertical column) and to the NMR structure of *T*. *brucei* Tb24 (right vertical column). (A) the predicted model of *T*. *congolense* calflagin (middle vertical column) exhibits four EF-hands motifs. The EF-hands (EF1, EF2, EF3 and EF4) are colored green, salmon, cyan, yellow, accordingly. Left panel: structural alignment of *T*. *congolense* (grey) over *T*. *cruzi* FCaBP (green). Right panel: *T*. *congolense* calflagin structure aligned over *T*. *brucei* Tb24 (magenta). (B) Surface representation of the three calflagin models overlapping panel A, showing exposed hydrophobic (gray), basic (blue) and acidic (red) respectively. (C) 180° rotation of B in the *y-axis*. Black arrows pointing towards putative epitopes and numbered according to their respective α-helix location.

To better understand how mAb Tc6/42.6.4 selectively recognizes *T*. *congolense* calflagin compared to homologues from *T*. *cruzi* (FCaBP) and *T*. *brucei* (Tb24) we generated a high confidence homology model of *T*. *congolense* calflagin using the I-TASSER server (Fig 6; [23]). Consistent with the high degree of shared sequence identity, the resulting *T*. *congolense* calflagin model superimposes well with calflagins from *T*. *cruzi* [18] (PDB code 3CS1—RMSD of 0.6 Å over 187 Cα positions) and *T*. *brucei* (Tb24) [33] (PDB code 2LVV—RMSD of 4.1 Å over 195 Cα positions).

The overall predicted structure incorporates eight α-helices and four β-strands that fold into four EF-hand substructures (Fig 6): EF1 (residues 47–74, *green*); EF2 (residues 96–124, salmon); EF3 (residues 129–157, cyan); EF4 (residues 166–194, yellow). Intriguingly, the 12-residue Ca^2+^- binding loop of EF2 from *T*. *cruzi* FCaBP [18] is loosely structured and adopts an unusual conformation that is unlikely to coordinate Ca^+2^. It is suggested that the loop is unstructured due to the presence of G109 at position-3 in the binding loop, which lacks the required acidic side chain therefore is unable to coordinate the Ca^2+^ ion at this key position. *T*. *congolense* EF2 shares the equivalent residue G107 at position-3 in addition to G105 at position-1 and is therefore also unlikely to coordinate Ca^2+^ at this position. Moreover, it is suggested that the Ca^+2^-free, closed conformation formed by EF1 and EF2 prevents Ca^2+^- binding at EF1. It is also suggested that the C66 at position-9 in the EF-1 loop is unable to form a hydrogen bond with E69, therefore might destabilize the binding loop structure and prevent Ca^+2^-binding at EF1. *T*. *congolense* calflagin incorporates S64 in the equivalent position, which is unable to form a hydrogen bond with E67 in the predicted model, which likely results in destabilization of the EF1 loop and restricted Ca^+2^-binding. The EF3 and EF4 substructures of the *T*. *cruzi* calflagins adopt more favorable local conformations and consist of functional residues at the key positions to enable Ca^+2^ binding. The EF3 Ca^+2^-binding loop of *T*. *congolense* calflagin, which consists of residues D138, S140, D142 and E149 (equivalent to D140, S142, N144 and E151 from the *T*. *cruzi* FCaBP) are likely functionally able to coordinate a Ca^+2^. While the EF4 Ca^+2^-binding loop incorporating residues D175, N177, T179 and E186 (equivalent to D177, N179, T181 and E188 from *T*. *cruzi* FCaBP) are also likely to coordinate Ca^+2^ in a similar geometry to *T*. *cruzi* FCaBP EF4. The conserved structural features of the EF3 and EF4 loops are consistent with the ability of *T*. *congolense* calflagin to bind Ca^+2^ as demonstrated here with its calcium dependent localization in the parasite.

### Putative epitope for the monoclonal antibody Tc6/42.6.4

In addition to the global structural characteristics of the model, the overlaid structures enabled a more thorough analysis of variations in surface chemistry and local topology. Calculation of surface charges reveals that calflagin from *T*. *cruzi* and *T*. *congolense* show comparable acidic and basic distributions while *T*. *brucei* calflagin displays a more acidic surface. These calculations are consistent with the calculated isoelectric points (pI) of 5.29, 4.9 and 4.67 for *T*. *congolense* calflagin, *T*. *cruzi* FCaBP and *T*. *brucei* Tb24, respectively. However, there do not appear to be localized charged regions that are specific to *T*. *congolense*. Analyzing the representative sequences in the context of the structures reveals that surface-exposed, non-conserved residues are not clustered but rather broadly distributed with many of the more divergent sequences mapping to surface loops. While this does make predicting the epitope for mAb Tc6/42.6.4 difficult, a comprehensive analysis of surface chemistry and local architecture allowed us to identify four regions that are most plausible to form linear epitopes on the surface of *T*. *congolense* calflagin. These include the surface-exposed regions of α-helices 2 (residues 42–47), 4 (residues 97–101), 6 (151–154) and 7 (168–174). Attempts to localize the epitopes to any of these regions by peptide mapping after trypsin- or Lys-C-cleavage or by digestion protection experiments after binding recombinant antigen with the mAb Tc6/42.6.4 were unsuccessful, presumably due to epitope cleavage or alteration of the topographically assembled epitope. No binding was seen in indirect ELISAs with small synthetic peptide sequences as solid-phase adsorbed antigen.

### Biochemical characterization of mAb Tc6/42.6.4

The heavy and light chain genes from the hybridoma secreting mAb Tc6/42.6.4 were isolated and sequenced. The sequences were most similar to the germline antibody genes IGHV1S52*01 and IGKV8-27*01 (S2 Appendix). MAb Tc6/42.6.4 was also tested in SPR assays using purified, recombinant calflagin as analyte for determination of binding kinetics. The mAb bound strongly to calflagin with a dissociation constant (KD) of 18 nM, an on-rate (ka) of 6.08e4 M^-1^ s^-1^ and an off-rate (kd) of 1.12e-3 s^-1^. The binding curves are shown in S3 Appendix.

### Derivation and testing of new anti-calflagin mAbs

To further study the epitopes of *T*. *congolense* calflagin, new anti-calflagin mAbs were generated using recombinant antigen as the immunogen. Of more than 1000 hybridoma supernatants screened, seven newly derived mAbs were shown to bind to recombinant *T*. *congolense* calflagin and also showed reactivity with *T*. *congolense* lysate by ELISA, presumably binding to the native calflagin. Two of the new mAbs also bound to an antigen in *T*. *b*. *brucei* PCF lysates, presumably calflagin. These results indicate that these mAbs recognize an epitope that is shared between the *T*. *b*. *brucei* and *T*. *congolense* calflagins. Thus both shared and *T*. *congolense*-specific epitopes are present in the recombinant *T*. *congolense* calflagin.

### Measurement of anti-calflagin antibodies in cattle sera

**Fig 7:**
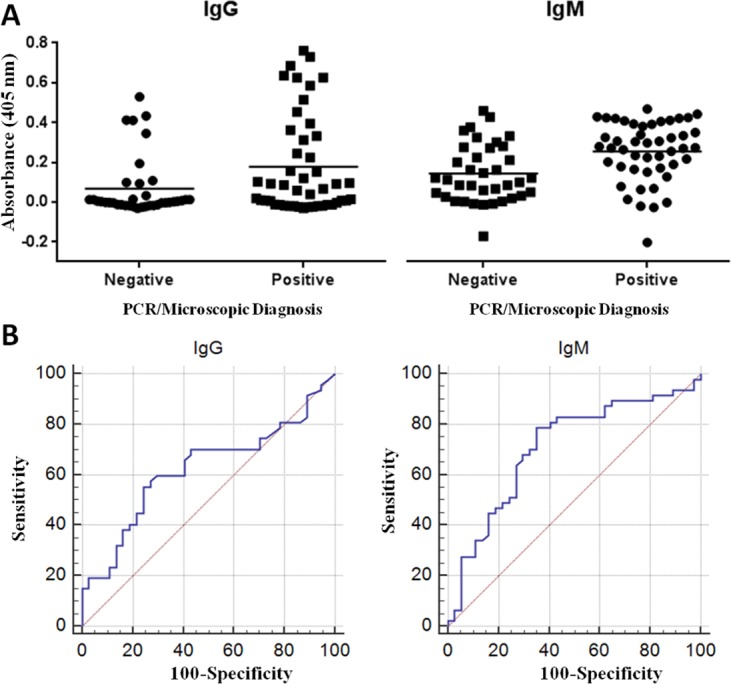
Calflagin-based serodiagnosis of trypanosome infections in Ugandan cattle. (A) ELISA signal intensities resulting from bovine IgG and IgM antibodies binding to solid-phase adsorbed recombiant *T*. *congolense* calflagin. All values were normalized against signals elicited from plasma of laboratory raised, trypanosome negative calves. (B) ROC curves generated from the serodiagnostic ELISA results. Areas under curve for IgG and IgM were 0.623 and 0.709 respectively. For reference, an area under the curve of 1.00 equates to a test with 100% sensitivity and specificity, whereas an area under the curve of 0.500 indicates that a test has no value in differentiating between the binary population.

Sera from cattle living in trypanosome/tsetse endemic areas of Uganda and from laboratory raised cattle that were not exposed to trypanosomes were tested for the presence of IgG and IgM anti-calflagin antibodies by indirect ELISA (Fig 7A and S4 Appendix). Whether or not cattle were infected with trypanosomes was determined by detection of parasites in blood using light microscopy and by detection of trypanosome-specific PCR products. PCR product could not be detected in 9 parasite positive samples, while 5 apparently parasite negative samples had signals for *T*. *congolense* and *T*. *brucei*. If an animal tested parasite positive by light microscopy and/or PCR, it was considered trypanosome-infected. By these criteria, of the 84 cattle tested, 47 animals were infected and 37 were uninfected. Samples from the three uninfected calves housed at Makerere University showed no observable parasites and yielded no trypanosome-specific PCR products. Calflagin's serodiagnostic performance is illustrated by ROC curves (reviewed in [29]). Measurement of calflagin-specific IgG and IgM yielded area under the curve (AUC) of 0.623 and 0.709 respectively (Fig 7B).

## Discussion

Trypanosome-specific antibodies are useful for basic research and for their potential application, for example in monitoring the presence of parasites in tsetse for epidemiological studies and for diagnosis of infections in humans and their domestic animals. In this regard, the monoclonal antibody Tc6/42.6.4 is of interest since it is one of the few trypanosome species-specific mAbs described to date [9]. To further characterize this monoclonal antibody we have extended species- and life-cycle specificity analyses, determined its antigen binding kinetics and sequenced the antibody heavy and light chains. This was done, in part, to address recent calls for better characterization of antibody reagents in order to enhance their utility in research and application. Our results show that mAb Tc6/42.6.4 does not bind to molecules in the other member of the subgenus *Nannomonas* (*T*. *simiae*) but recognizes molecules in both savannah and Kilifi groups of *T*. *congolense*, thus is clearly *T*. *congolense*- specific. The antibody recognizes a protein of the same apparent molecular mass (26 kDa) in all four major life cycle stages of *T*. *congolense*, thus should be useful in detecting the parasites in the tsetse vector, in infected mammals and in culture forms *in vitro*. The antibody has been shown to work in ELISA, immunofluorescence assays, immunoblotting and immunoprecipitation assays and exhibits high avidity in SPR assays thus is of broad, general utility for laboratory use.

Many attempts were made over more than two decades to identify the antigen recognized by the *T*. *congolense*-specific mAb Tc6/42.6.4. The antigen appeared to be moderately abundant as judged by immunofluorescence observations of fixed, permeabilized whole parasites yet only a relatively faint protein band corresponding to immunoreactive material could be visualized by staining polyacrylamide gels. The protein appears to be highly immunogenic, since antibodies were produced against this molecule in several experiments after immunization of mice with whole parasites [9]. Previous attempts at identifying the antigen generally involved immunoenrichment followed by gas-phase Edman N-terminal sequencing from PVDF membranes and all failed, perhaps due to inadequate amounts of purified antigen or to blocking of N-terminal amino acid residues of the antigen. Ultimately, in the work reported here, a mass spectrometric method was successful in identifying the antigen after enrichment from parasite lysates using mAb Tc6/42.6.4. Based on the sequence of two contiguous peptides, the antigen was determined to be a trypanosome flagellar calcium-binding protein (also known as FCaBP or calflagin). Homologues of calflagin are found throughout the order *Trypanosomatidae* [19, 33–35]. Calflagin is di-acylated by palmitoylation and myristoylation of its N terminus and is thus predicted to be able to associate with the plasma membrane, with the highest concentration located along the flagellum [32]. Calcium binding is required for membrane localization of calflagin and when calcium chelators are added, these proteins become cytoplasmic [17, 32, 36]. This phenomenon was recapitulated through the immunofluorescence experiments described here, indicating that trypanosome calflagins behave similarly in different parasite species. The calcium dependent localization of calflagin to the flagellar membrane implies that its effect is directed at the flagellum, possibly involving the regulation of flagellar function, although a role as a calcium sensor cannot be discounted.

It is known that depletion of calflagin (by RNA interference) in *T*. *brucei* does not inhibit growth or motility *in vitro*, indicating that calflagin is not an essential component for flagellar activity [37]. However, these same calflagin depleted BSF parasites were markedly attenuated when used to infect mice. The mice experienced a reduced parasite burden and consequently survived longer than those infected with wild-type parasites. The reason for this attenuation remains unknown but provides tantalizing possibilities for interference with calflagin as a new drug target.

*T*. *cruzi* calflagin is highly immunogenic in humans and has been used since the 1990’s as an antigen for serodiagnosis of Chagas’ disease caused by *T*. *cruzi* [38, 39] and for monitoring efficacy of treatment following chemotherapy [40]. The results reported here showed that *T*. *congolense* calflagin is highly immunogenic in mice (the source of the mAbs) and that naturally infected cattle produce anti-calflagin antibodies as a result of trypanosome infection. However, the serodiagnostic results obtained using sera from naturally infected cattle were disappointing, although not necessarily surprising for animals resident in an endemic area under constant tsetse challenge. Noteworthy is that the diagnostic efficiency calculated here (AUC of 0.623 and 0.709 respectively for IgG and IgM) may be an underestimate since it is possible that some of the false positives are the result of animals being misclassified by microscopy and PCR as uninfected, due to low parasite burden at time of sampling. Alternatively, animals recently treated or self-cured of trypanosomes may still maintain antibody levels detectable by ELISA thereby falsely identifying them as infected. Conversely, false negatives could result from early stage infection before the cattle mounted an immune response. Testing of sera from experimentally infected cattle may offer better results but with less practical value for determining naturally occurring infections in the field. Calflagin still appears to be a promising antigen for diagnostic purposes however, and the new well-characterized tools developed during the work reported here (the recombinant *T*. *congolense* calflagin and the new species-specific and pan-specific anti- calflagin mAbs) will allow much more complete testing of calflagin as a serodiagnostic antigen and as a target for antigen detection tests which should be indicative of active infections. Whether or not the sensitivity of such antigen detection tests would be sufficient, since natural infections commonly show characteristic low parasitemias, is a subject of debate. In addition, development of antigen detection tests into a field format remains challenging.

## Supporting Information

S1 Appendix Gel electrophoresis and immunoblotting analysis of immunoenriched and recombinant *T*. *congolense* calflagin. Left upper panel: Immunoblot Figure showing the results of pull-down experiments using the *T*. *congolense*-specific mAb Tc6/42.6.4 and detection of immunoreactive and bands. The Figure shows the overlay of two images: the autoluminogram showing the immunoreactive bands and the PVDF membrane that had been stained with nigrosin after development of the autoluminogam. Lane 1: molecular mass standards with apparent masses shown on the extreme left. Lane 2: proteins enriched from *T*. *congolense* Il3000 PCF using mAb Tc6/42.6.4. Lane 3: *T*. *congolense* IL3000 PCF lysate. The right upper panel shows a Colloidal Coomassie Blue stained gel of *T*. *congolense* Il3000 PCF proteins. Lane 4: Molecular mass standards. Lane 5: Proteins enriched from *T*. *congolense* Il3000 PCF using mAb Tc6/42.6.4. The boxed band shows the portion of the gel that was excised for trypsin digestion and mass spectrometric analysis. Lane 6: *T*. *congolense* Il3000 PCF lysate. The lower panels shows the expression and detection of recombinant *T*. *congolense* calflagin in *E*. *coli*. The left lower panel shows immunoblot detection of recombinant calflagin using mAb Tc6/42.6.4. Lane 1. Molecular mass standards. Lane 2: *E*. *coli* transformed with calflagin in pET-24a, not induced. Lane 3: *E*. *coli* transformed with pET-24a, induced with IPTG. The right lowe panel shows a colloidal Coomassie Blue stained gel of *E*. *coli* lysates. Lane 1. Molecular mass standards. Lane 2: *E*. *coli* transformed with calflagin in pET-24a, not induced. Lane 3: *E*. *coli* transformed with pET-24a, induced with IPTG.
(TIF)

S2 Appendix DNA and protein sequences of the monoclonal antibody Tc6/42.6.4 heavy and light chains. (PDF)

S3 Appendix Multi-concentration analysis of binding kinetics of mAb Tc6/42.6.4 to *T*. *congolense* calflagin by surface plasmon resonance. Recombinant calflagin was injected over captured mAb Tc6/42.6.4 at concentrations of 62.5 nM, 125 nM, 250 nM and 500 nM (bottom to top curves respectively). Data were double referenced and fit globally using a 1:1 Langmuir binding model.
(TIF)

S4 Appendix Serodiagnostic ELISA data obtained using plasma of Ugandan cattle. Recombinant *T*. *congolense* calflagin was used as solid-phase adsorbed antigen.
(XLSX)

## References

[1] Eyford BA, Sakurai T, Smith D, Loveless B, Hertz-Fowler C, Donelson JE, et al. Differential protein expression throughout the life cycle of *Trypanosoma congolense*, a major parasite of cattle in Africa. Mol Biochem Parasitol. 2011; 177:116–125. 10.1016/j.molbiopara.2011.02.009 21354217 PMC3820035

[2] Hirumi H, Hirumi K. *In vitro* cultivation of Trypanosoma congolense bloodstream forms in the absence of feeder cell layers. Parasitology. 1991; 102: 225–236. 1852490 10.1017/s0031182000062533

[3] Beecroft RP, Roditi I, Pearson TW. Identification and characterization of an acidic major surface glycoprotein from procyclic stage *Trypanosoma congolense*. Mol Biochemical Parasitol. 1993; 61: 285–294. 10.1016/0166-6851(93)90074-8 7903427

[4] Bütikofer P, Vassella E, Boschung M, Renggli CK, Brun R, Pearson TW, et al. Glycosylphosphatidylinositol-anchored surface molecules of *Trypanosoma congolense* insect forms are developmentally regulated in the tsetse fly. Mol Biochem Parasitol. 2002; 119: 7–16. 11755181 10.1016/s0166-6851(01)00382-6

[5] Sakurai T, Sugimoto C, Inoue N. Identification and molecular characterization of a novel stage-specific surface protein of *Trypanosoma congolense* epimastigotes. Mol Biochem Parasitol. 2008; 161: 1–11. 10.1016/j.molbiopara.2008.05.003 18571746

[6] Tonkin ML, Workman SD, Eyford BA, Loveless BC, Fudge JL, Pearson TW, et al. Purification, crystallization and X-ray diffraction analysis of *Trypanosoma congolense* insect-stage surface antigen (TcCISSA). Acta Crystallogr F. 2012; 68: 1503–1506. 10.1107/S1744309112042686 PMC3509974 23192033

[7] Nantulya VM, Musoke AJ, Rurangirwa FR, Saigar N, Minja SH. Monoclonal antibodies that distinguish *Trypanosoma congolense*, *T*. *vivax* and *T*. *brucei*. Parasite Immunol. 1987; 9: 421–431. 3306569 10.1111/j.1365-3024.1987.tb00520.x

[8] Masake RA, Nantulya VM. Sensitivity of an antigen detection enzyme immunoassay for diagnosis of *Trypanosoma congolense* infections in goats and cattle. J Parasitol. 1991; 77: 231–236. 2010855

[9] Parish NM, Morrison WI, Pearson TW. Identification of an antigen specific to *Trypanosoma congolense* by using monoclonal antibodies. J Immunol. 1985; 134: 593–597. 3880577

[10] Fish WR, Muriuki CW, Muthiani AM, Grab DJ, Lonsdale-Eccles JD. Disulfide bond involvement in the maintenance of the cryptic nature of the cross-reacting determinant of metacyclic forms of *Trypanosoma congolense*. Biochemistry. 1989; 28: 5415–5421. 2476173 10.1021/bi00439a015

[11] Paling RW, Moloo SK, Jenni L. *Trypanosoma congolense*: host responses following tsetse transmitted infection of Kilifi isolates in goats. Exp Parasitol. 1987; 63: 279–287. 3582569 10.1016/0014-4894(87)90174-3

[12] Zweygarth E, Röttcher D. The occurrence of *Trypanosoma (Nannomonas) simiae* in the cerebrospinal fluid (CS) of domestic pigs. Parasitol Res. 1987; 73: 479–480. 3658977 10.1007/BF00538209

[13] Brun R, Schönenberger M. Cultivation and *in vitro* cloning or procyclic culture forms of *Trypanosoma brucei* in a semi-defined medium. Acta Tropica. 1979; 36: 289–292. 43092

[14] Neuhoff V, Arold N, Taube D, Ehrhardt W. Improved staining of proteins in polyacrylamide gels including isoelectric focusing gels with clear background at nanogram sensitivity using Coomassie Brilliant Blue G-250 and R-250. Electrophoresis. 1988; 9: 255–262. 2466658 10.1002/elps.1150090603

[15] Haddow JD, Poulis B, Haines LR, Gooding RH, Aksoy S, Pearson TW. Identification of major soluble salivary gland proteins in teneral *Glossina morsitans morsitans*. Insect Biochem Molec. 2002; 32: 1045–1053. 10.1016/s0965-1748(02)00042-5 12213241

[16] Bains J, R. L, K.G. T, M.J. B. Elucidating the reaction mechanism of the benzoate oxidation pathway encoded aldehyde dehydrogenase from *Burkholderia xenovorans* LB400. Protein Science. 2011; 20: 1048–1059. 10.1002/pro.639 21495107 PMC3104234

[17] Pinto APA, Campana PT, Beltramini LM, Silber AM, Araújo APU. Structural characterization of a recombinant flagellar calcium-binding protein from *Trypanosoma cruzi*. Biochim Biophys Acta. 2003; 1652: 107–114. 14644046 10.1016/j.bbapap.2003.08.008

[18] Wingard JN, Ladner J, Vanarotti M, Fisher AJ, Robinson H, Buchanan KT, et al. Structural insights into membrane targeting by the flagellar calcium-binding protein (FCaBP), a myristoylated and palmitoylated calcium sensor in *Trypanosoma cruzi*. J Biol Chem. 2008; 283: 23388–23396. 10.1074/jbc.M803178200 18559337 PMC2516990

[19] Buchanan KT, Ames JB, Asfaw SH, Wingard JN, Olson CL, Campana PT, et al. A flagellum-specific calcium sensor. J Biol Chem. 2005; 280: 40104–40111. 16148003 10.1074/jbc.M505777200

[20] Tolson DL, Turco SJ, Beecroft RP, Pearson TW. The immunochemical structure and surface arrangement of *Leishmania donovani* lipophosphoglycan determined using monoclonal antibodies. Mol Biochem Parasitol. 1989; 35: 109–118. 2475775 10.1016/0166-6851(89)90113-8

[21] Schlaeppi K, Deflorin J, Seebeck T. The major component of the paraflagellar rod of *Trypanosoma brucei* is a helical protein that is encoded by two identical, tandemly linked genes. J Cell Biol. 1989; 109: 1695–1709. 2793936 10.1083/jcb.109.4.1695 PMC2115804

[22] Pope ME, Soste MV, Eyford BA, Anderson NL, Pearson TW. Anti-peptide antibody screening: Selection of high affinity monoclonal reagents by a refined surface plasmon resonance technique. J Immunol Methods. 2009; 341: 86–96. 10.1016/j.jim.2008.11.004 19041872

[23] Roy A, Kucukural A, Zhang Y. I-TASSER: a unified platform for automated protein structure and function prediction. Nat Protocols. 2010; 5:725–738. 10.1038/nprot.2010.5 20360767 PMC2849174

[24] DeLano WL. The PyMOL Molecular Graphics System. San Carlos, USA: DeLano Scientific; 2002.

[25] Holm L, Park J. DaliLite workbench for protein structure comparison. Bioinformatics. 2000; 16: 566–567. 10980157 10.1093/bioinformatics/16.6.566

[26] Woo PTK. The haematocrit centrifuge technique for the diagnosis of African trypanosomiasis. Acta Trop. 1970; 27: 384–386. 4396363

[27] Njiru ZK, Constantine CC, Guya S, Crowther J, Kiragu JM, Thompson RCA, et al. The use of ITS1 rDNA PCR in detecting pathogenic African trypanosomes. Parasitol Res. 2005; 95:186–192. 15619129 10.1007/s00436-004-1267-5

[28] Cox A, Tilley A, McOdimba F, Fyfe J, Eisler M, Hide G, et al. A PCR based assay for detection and differentiation of African trypanosome species in blood. Exp Parasitol. 2005; 111:24–29. 16054487 10.1016/j.exppara.2005.03.014

[29] Florkowski CM. Sensitivity, Specificity, Receiver-Operating Characteristic (ROC) Curves and Likelihood Ratios: Communicating the Performance of Diagnostic Tests. Clin Biochem Rev. 2008; 29: S83–S87. 18852864 PMC2556590

[30] Loveless BC, J.W. M, Sakurai T, Inoue N, Razavi M, Pearson TW, et al. Structural Characterization and Epitope Mapping of the Glutamic Acid/Alanine-rich Protein from *Trypanosoma congolense*: Defining Assembly of the Parasite Cell Surface. J Biol Chem. 2011; 286: 20658–20665. 10.1074/jbc.M111.218941 21471223 PMC3121512

[31] Edgar RC. MUSCLE: a multiple sequence alignment method with reduced time and space complexity. BMC Bioinformatics. 2004; 5:113 15318951 10.1186/1471-2105-5-113 PMC517706

[32] Godsel LM, Engman DM. Flagellar protein localization mediated by a calcium-myristoyl/palmitoyl switch mechanism. EMBO J. 1999; 18: 2057–2065. 10205160 10.1093/emboj/18.8.2057 PMC1171290

[33] Xu X, Olson CL, Engman DM, Ames JB. NMR structure of the calflagin Tb24 flagellar calcium binding protein of *Trypanosoma brucei*. Protein Science. 2012; 21: 1942–1947. 10.1002/pro.2167 23011904 PMC3575923

[34] Maldonado RA, Linss J, Thomaz N, Olson CL, Engman DM, Goldenberg S. Homologues of the 24-kDa flagellar Ca^2+^-binding protein gene of *Trypanosoma cruzi* are present in other members of the Trypanosomatidae family. Exp Parasitol. 1997; 86: 200–205. 9225770 10.1006/expr.1997.4159

[35] Engman DM, Krause KH, Blumin JH, Kim KS, Kirchhoffd LV, Donelson JE. A Novel Flagellar Ca^2+^-binding protein in Trypanosomes. J Biol Chem. 1989; 264:18627–18631. 2681200

[36] Maldonado RA, Mirzoeva S, Godsel LM, Lukas TJ, Goldenberg S, Watterson DM, et al. Identification of calcium binding sites in the trypanosome flagellar calcium-acyl switch protein. Mol Biochem Parasitol. 1999; 101: 61–70. 10413043 10.1016/s0166-6851(99)00055-9

[37] Emmer BT, Daniels MD, Taylor JM, Epting CL, Engman DM. Calflagin Inhibition Prolongs Host Survival and Suppresses Parasitemia in *Trypanosoma brucei* Infection. Eukaryot Cell. 2010; 9: 934–942. 10.1128/EC.00086-10 20418379 PMC2901653

[38] Godsel LM, Tibbetts RS, Olson CL, Chaudoir BM, Engman DM. Utility of Recombinant Flagellar Calcium-Binding Protein for Serodiagnosis of *Trypanosoma cruzi* Infection. J Clin Microbiol. 1995; 33: 2082–2085. 7559952 10.1128/jcm.33.8.2082-2085.1995 PMC228339

[39] Marcipar IS, Roodveldt C, Corradi G, Cabeza ML, Brito MEF, Winter LMF, et al. Use of Full-Length Recombinant Calflagin and Its C Fragment for Improvement of Diagnosis of *Trypanosoma cruzi* Infection. J Clin Microbiol. 2005; 43: 5498–5503. 16272476 10.1128/JCM.43.11.5498-5503.2005 PMC1287826

[40] Sosa E.S. SE, Ruiz AM, Velazquez E, Porcel BM, Yampotis C. Efficacy of chemotherapy with benznidazole in children in the indeterminate phase of Chagas' disease. Am J Trop Med Hyg. 1998; 59: 526–529. 9790423 10.4269/ajtmh.1998.59.526

