# Supplementary figures and images for: Characterization of Calflagin, a Flagellar Calcium-Binding Protein from Trypanosoma congolense
Source: PLoS Negl Trop Dis. 2016 Apr 7;10(4):e0004510. doi: 10.1371/journal.pntd.0004510 (PMC4824491; doi:10.1371/journal.pntd.0004510)

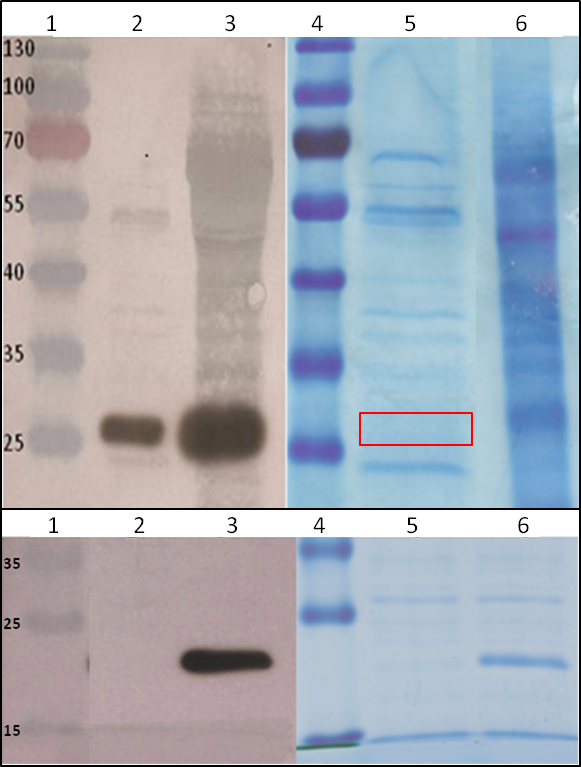

Supplement: S1 Appendix — Left upper panel: Immunoblot Figure showing the results of pull-down experiments using the T. congolense-specific mAb Tc6/42.6.4 and detection of immunoreactive and bands. The Figure shows the overlay of two images: the autoluminogram showing the immunoreactive bands and the PVDF membrane that had been stained with nigrosin after development of the autoluminogam. Lane 1: molecular mass standards with apparent masses shown on the extreme left. Lane 2: proteins enriched from T. congolense Il3000 PCF using mAb Tc6/42.6.4. Lane 3: T. congolense IL3000 PCF lysate. The right upper panel shows a Colloidal Coomassie Blue stained gel of T. congolense Il3000 PCF proteins. Lane 4: Molecular mass standards. Lane 5: Proteins enriched from T. congolense Il3000 PCF using mAb Tc6/42.6.4. The boxed band shows the portion of the gel that was excised for trypsin digestion and mass spectrometric analysis. Lane 6: T. congolense Il3000 PCF lysate. The lower panels shows the expression and detection of recombinant T. congolense calflagin in E. coli. The left lower panel shows immunoblot detection of recombinant calflagin using mAb Tc6/42.6.4. Lane 1. Molecular mass standards. Lane 2: E. coli transformed with calflagin in pET-24a, not induced. Lane 3: E. coli transformed with pET-24a, induced with IPTG. The right lowe panel shows a colloidal Coomassie Blue stained gel of E. coli lysates. Lane 1. Molecular mass standards. Lane 2: E. coli transformed with calflagin in pET-24a, not induced. Lane 3: E. coli transformed with pET-24a, induced with IPTG. (TIF) [file pntd.0004510.s001.tif]

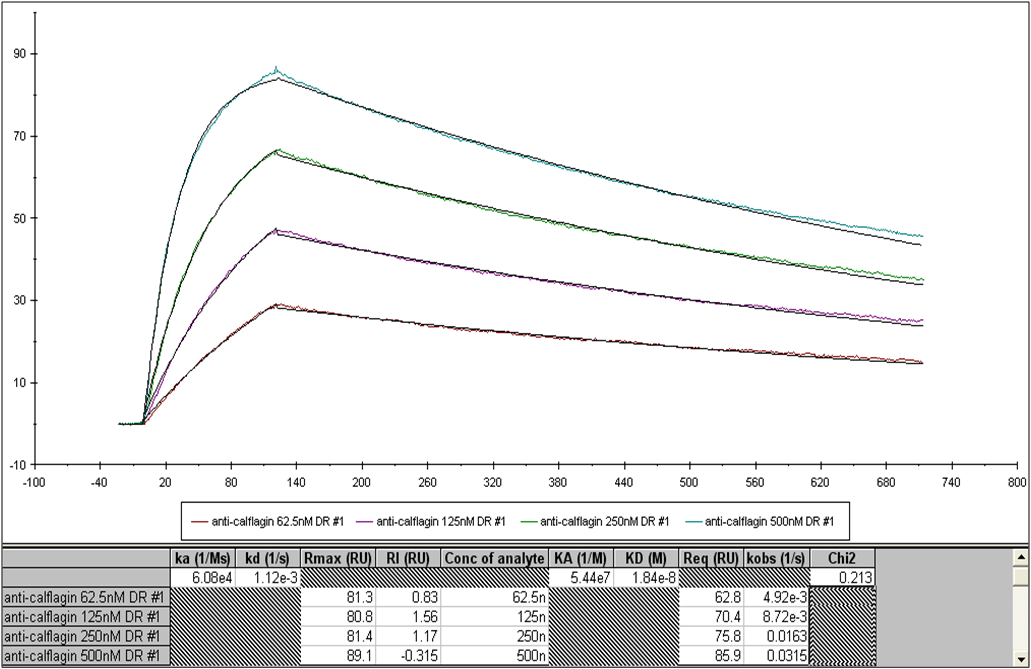

Supplement: S3 Appendix — Recombinant calflagin was injected over captured mAb Tc6/42.6.4 at concentrations of 62.5 nM, 125 nM, 250 nM and 500 nM (bottom to top curves respectively). Data were double referenced and fit globally using a 1:1 Langmuir binding model. (TIF) [file pntd.0004510.s003.tif]
