# Supplementary material for: Characterization of Calflagin, a Flagellar Calcium-Binding Protein from Trypanosoma congolense
Source: PLoS Negl Trop Dis. 2016 Apr 7;10(4):e0004510. doi: 10.1371/journal.pntd.0004510 (PMC4824491; doi:10.1371/journal.pntd.0004510)
Supplement: S2 Appendix — (PDF) [file pntd.0004510.s002.pdf]

**Tc6/42.6.4, Heavy chain, V region, $IgG_{2b}$**
**Closest Germline gene =** IGHV1S52*01

**Protein:**
...WNFEVQLQESGAELVRPGTSVKVSCKASGYVLRNYLIEWVKQRPGQGLEWIGVINPGSGNT
DYNEKFKGKATLTADKSSRTAYMQLSSLTSDDSAVYFCARGLWGYFDYWGQGTTLTVSSAKTTP
PSVYPLAPGXGDTTV...

**DNA:**
...TGGAATTTTGAGGTGCAGCTGCAGGAGTCTGGAGCTGAACTGGTAAGGCCTGGGACTTCAG
TGAAGGTGTCCTGCAAGGCTTCTGGATACGTCCTCAGAAATTACTTGATAGAGTGGGTAAAGCA
GAGGCCTGGACAGGGCCTTGAGTGGATTGGAGTGATTAATCCTGGAAGTGGCAATACTGACTAC
AATGAGAAGTTCAAGGGCAAGGCAACACTGACTGCAGACAAATCCTCCAGAACTGCCTACATGC
AGCTCAGCAGTCTGACATCTGATGACTCTGCGGTTTATTTCTGTGCAAGAGGACTTTGGGGTTA
TTTTGACTACTGGGGCCAAGGCACCACTCTCACAGTCTCCTCAGCCAAAACAACACCCCCATCA
GTCTATCCACTGGCCCCTGGGTGNGGAGATACAACTGTC....

**Tc6/42.6.4, Light chain, V region, Kappa**
**Closest Germline gene =** IGKV8-27*01

**Protein:**
...LLFWVSGTCGNIMMTQSPSSLAVSAGEKVTMSCKSSRSVLYSSNQKNYLAWYQQKPGQSPK
LLIYWASTRESGVPDRFTGSGSGTDFTLTISSVQVEDLAVYYCHQYLSSRTFGGXP...

**DNA:**
...TTGCTGTTCTGGGTATCTGGTACCTGTGGGAACATTATGATGACACAGTCGCCATCATCTC
TGGCTGTGTCTGCAGGAGAAAAGGTCACTATGAGCTGTAAGTCCAGTCGAAGTGTTTTATACAG
TTCAAATCAGAAGAACTACTTGGCCTGGTACCAGCAGAAACCAGGGCAGTCTCCTAAACTGCTG
ATCTACTGGGCATCCACTAGGGAATCTGGTGTCCCTGATCGCTTCACAGGCAGTGGATCTGGGA
CAGATTTTACTCTTACCATCAGCAGTGTTCAAGTTGAAGACCTGGCAGTTTATTACTGTCATCA
ATACCTCTCCTCGCGGACGTTCGGTGGNNNACCA.....
